# A theoretical probe into the separation of CO_2_/CH_4_/N_2_ mixtures with polysulfone/polydimethylsiloxane–nano zinc oxide MMM

**DOI:** 10.1038/s41598-023-36051-1

**Published:** 2023-06-12

**Authors:** Reza Soleimani, Amir Hossein Saeedi Dehaghani

**Affiliations:** 1 Faculty of Chemical Engineering, Tarbiat Modares University, P.O. Box 14115-143, Tehran, Iran; 2 Department of Petroleum Engineering, Faculty of Chemical Engineering, Tarbiat Modares University, P.O. Box 14115-143, Tehran, Iran

**Keywords:** Theoretical chemistry, Molecular dynamics, Chemical engineering, Pollution remediation, Sustainability

## Abstract

In the current investigation, molecular dynamics (MD) and Grand Canonical Monte Carlo (GCMC) simulation as remarkable and competent approaches have been employed for understanding structural and transport properties of MMMs in the realm of gas separation. The two commonly used polymers i.e. polysulfone (Psf) and polydimethylsiloxane (PDMS) as well as zinc oxide (ZnO) nanoparticle (NP) were used to carefully examine the transport properties of three light gasses (CO_2_, N_2_ and CH_4_) through simple Psf, Psf/PDMS composite loaded by different amounts of ZnO NP. Also, the fractional free volume (FFV), X-ray diffraction (XRD), glass transition temperature (T_g_), and Equilibrium density were calculated to scrutinize the structural characterizations of the membranes. Moreover, the effect of feed pressure (4–16 bar) on gas separation performance of simulated MMMs was investigated. Results obtained in different experiments showed a clear improvement in the performance of simulated membranes by adding PDMS to PSf matrix. The selectivity of studied MMMs was in the range from 50.91 to 63.05 at pressures varying from 4 to 16 bar for the CO_2_/N_2_ gas pair, whereas the corresponding value for CO_2_/CH_4_ system was found to be in the range 27.27–46.24. For 6 wt% ZnO in 80%PSf + 20%PDMS membrane, high permeabilities of 78.02, 2.86 and 1.33 barrers were observed for CO_2_, CH_4_ and N_2_ gases, respectively. The 90%PSf + 10%PDMS membrane with 2% ZnO had a highest CO_2_/N_2_ selectivity value of 63.05 and its CO_2_ permeability at 8 bar was 57 barrer.

## Introduction

Carbon dioxide (CO_2_) is the most hazardous and prevalent greenhouse gas (GHG) that its accumulation in the environment drives global climate change^1^. Worldwide CO_2_ emissions have been projected to experience a sharp increase from 28,051 million metric tons (MMT) to about 42,325 MMT from 2005 to 2030^2^. Besides, the global consumption of natural gas is projected to increase from 95 trillion cubic feet in 2003 to 182 trillion cubic feet in 2030^3,4^. Beyond that, high levels of CO_2_ and Nitrogen (N_2_) gases were found as the major contaminants in the natural feed gas which must be reduced below 2–3% to meet pipeline specifications^1,5^. Additionally, CO_2_ lowers the calorific value of natural gas, and also causes severe corrosion problems in oil and gas pipelines as well as storage systems in the presence of water^1,6,7^. Therefore, purification and recovery of CO_2_ from flue gas are of great interest from the environmental and energy point of view. In this respect, using competent approaches, CO_2_ must be separated from natural gas before it enters the pipelines or released into the air.

The current conventional methods for the separation of CO_2_ from other gas molecules majorly encompass physical/chemical absorption, pressure swing adsorption, or cryogenic distillation^8^. Although, these conventional technologies have proven favorable separation performance, they still suffer from severe drawbacks such as low CO_2_ loading capacity, high equipment corrosion, high energy consumption^4^, high solution circulation rate and solution degradation, high capital costs for equipment, high circulation rate, high sulfur outlet content and undesired absorption of higher hydrocarbons with CO_2_ which results in hydrocarbon losses^9,10^. On the other hand, membranes as advanced technologies have been proven their promising role for gas separation. Simplicity of operation and installation, feasibility under mild conditions, smaller footprint and flexibility of operation due to compactness of modules with huge reduction in consumption of electricity and fuel, no need for extra agents and/or chemical, continuous mode of operation with partial or complete recycle of retentate/permeate, possibility of integration with other separation units to constitute effective hybrid processes for achieving improved economy and desired purity levels are some of the advantages of membrane separation processes^8,11,12^. Also, membranes can be “tailored” to adapt to a specific separation task^3^. Among the various kinds of membranes, polymeric membranes have received significant attention, mainly because they offer many desired properties such as excellent processing ability, low expense and high mechanical strength. Nevertheless, a limit in the trade-off between gas selectivity and permeability for these widely used membranes, has been identified as aptly shown by Robeson s “upper bound”^13–15^. Moreover, their poor thermal and chemical stability and the plasticization (related to CO_2_ concentration) also hamper the expansion of their province. Higher permeability and selectivity accompanied by good chemical and thermal resistance can be achieved by inorganic membranes such as zeolite^16^, mesoporous silica, carbon molecular sieve^17^, metal–organic frameworks (MOFs)^18,19^, alumina^20^ and carbon nanotubes, but their fabrication is challenging and usually involves higher cost which thwarted their employment in large-scale industrial applications^13,21,22^. The constraint of both polymeric and inorganic membranes have in turn changed the concentration of researchers toward mixed matrix membranes (MMMs) which are fabricated by introducing inorganic particles at nanometer level into polymer matrix. They integrate the advantages of both excellent selectivity characteristic of inorganic materials and economical processing capabilities together with the acceptable mechanical properties of polymers^21,23–28^. In this regard, the preparation of high-quality MMMs with the ideal morphology has been greatly challenged by the formation of defects or voids in the membranes interfaces which is usually caused by poor compatibility of organic materials as fillers with the polymer matrix. This type of voids which are morphologically known as “sieve-in-a-cage” act as additional channels that allow non-selective gas transport; therefore the selectivity of the whole membrane reduces^15,29–31^. Hence, careful selection of both inorganic fillers and polymers is essential to attain a successful fabrication of MMM with acceptable gas separation performance. It is worth pointing that, different filler and polymer materials have been recently employed for the fabrication of MMMs in the realm of CO_2_/CH_4_ separation^22,32,33^.

The fabrication of new MMMs for a particular separation system by experiment measurements is laborious process and often awkward, time-consuming, and expensive. On the other hand, with the dramatic advances in mathematical algorithms using computational practices, simulation has appeared an effective tool in material science and engineering^34–39^. In this sense, in depth analyses of the microscopic mechanisms which affect membrane structures and properties quantitatively and qualitatively by computer simulation and use of molecular models and simulation techniques is requisite. Simulation at microscopic and mesoscopic level can offer detailed insight into the fundamentals of the membrane fabrication and its features. Insights given by simulation are valuable for the proper design of the separation, characterization, screening, and for the development of novel membranes with improved performance^40^.

In the last few decades, molecular simulation (MS) has been increasingly employed to predict the behavior of systems on an atomic scale with a reasonable degree of accuracy and reliability^41–43^. Besides, Grand Canonical Monte Carlo (GCMC)^44^ and molecular dynamics (MD) simulation methods have been widely utilized to obtain new insight on the transport behavior of gas molecules within polymeric membranes (i.e. MMMs) as well as considering various structural characteristics such as fractional free volume (FFV), glass transition temperature (T_g_), radial distribution function (RDF), and X-ray diffraction (XRD), Wide-angle XRD (WAXD) of the system^40,45–47^. On this subject, Asghari et al.^48^ simulated the experiment results of a novel chitosan/silica MMMs filled with 10 wt% content of TEOS and APTEOS using MD and GCMC methods. The XRD test was accomplished to investigate the crystallinity of the simulated membranes in which the obtained results proved that membranes loaded with APTEOS indicated lower crystallinity compare to membranes with TEOS loading. To this end, the T_g_ of membranes containing APTEOS and TEOS was reported around 162 and 161 °C, respectively. In another study^49^, the influence of poly(ether block-6-amid) (PEBA) 1657 and zeolite 13× contents deposited onto a PSf/Polyethylene (PE) layer, on gas separation were evaluated. Both permeability and selectivity values of simulated membranes were examined using N_2_, CO_2_ and CH_4_ gas molecules. It was reported that, having coated a PDMS skin layer on the surface of PEBA-zeolite PSf/PE MMM resulted in 153% and 18.24% increase in CO_2_/N_2_ and CO_2_/CH_4_ selectivity, respectively. Also, they^50^ studied the effect of nanomaterials shape (nanorod and nanosphere) on gas separation performance by simulating PEBA 1657/ZnO MMMs. Beyond that, structural properties were measured by applying FFV and WAXD analyses. In another study, Golzar et al.^51^ investigated the transport properties CO_2_, CH_4_, N_2_, and O_2_ gas molecules through MMMs using MD and GCMC methods. Pristine and functionalized single wall carbon nanotube and multi wall carbon nanotube were embedded into the pure PIM-1 to study the CNT dispersion in the polymer matrix and its performance improvement. Also, the same authors^52^ studied the separation process of acid gas molecules such as H_2_S and CO_2_ from N_2_ and CH_4_ using simple polymeric membranes including PEBA-1657, poly (acrylonitrile) (PAN) and poly (trimethylsilyl) propyne (PTMSP) and zeolitic imidazole framework (ZIF) with various nanofiller loading in which both MD and GCMC simulation methods were applied. It was reported that, the MMMs incorporated with 2 wt% of the functionalized CNT particles indicated better performance for the CO_2_ separation compare to other simulated membranes. In order to consider the transport properties of simple PEBA polymer, simple Psf polymer, and PEBA/Psf composites loaded by ZIF-90 particles, three different gas molecules (CO_2_, CH_4_ and N_2_) were hired^53^. It was proved that Psf addition to PEBA polymer matrix, resulted in significant increase in the selectivity of CO_2_/CH_4_ and CO_2_/N_2_ gases. Meanwhile, loading ZIF-90 particles into the polymer matrix led to an upward trend for gas permeability of PEBA/Psf composites. Thus, the nanomaterials content loaded in polymer matrix, type of polymer and nano content, the process of membrane preparation, operating temperature and pressure, and the utilized solvents and anti-solvents are the most influential factors on the performance of membranes in the realm of gas separation.

In current study, eight different MMMs composed of blended PDMS/Psf polymers loaded with ZnO nanoparticles (NPs) as fillers have been simulated to scrutinize structural and transport properties of this novel kind of MMMs. Transport properties of three different gas molecules (CO_2_, CH_4_ and N_2_) through the simulated MMMs have been well investigated and discussed. Herein, surface topography, morphology, sorption and diffusion of gas molecules, the membrane crystallinity, and other structural and transport properties of this MMM have been studied. Furthermore, all the simulation results have been extracted and compared with experimental result to examine the reliability of current simulation which is proven that both results are consistent in approach.

## Simulation theory

### Force field

A force field consists of a series of potential functions and numerical parameters to explain the interaction potential. In the past, a number of these power fields have been developed for a variety of systems. For example, the force field of the Molecular Mechanics (MM) force field can be used for organic compounds, free radicals, and ions^54^. Another force field called AMBER is suitable for proteins, nucleic acids and polysaccharides^55^. Moreover, there are some promising, comprehensive and more complicated force fields which can be used to measure complex properties of materials like molecular structure, spectrum, and adaptations. These parameters have been obtained using a combination of mechanical quantum computing and laboratory data. PCFF, CVFF, Deriding, Universal, and COMPASS are the main and the most commonly used force field. In present article, the COMPASS force field has been utilized not just because of covering all the molecular interactions, but because COMPASS is a promising force field that supports atomistic simulations of condensed phase materials and represent the state-of-the-art force field technology^56^. COMPASS force field is able to predict the properties of a broad range of systems with high accuracy. Its main aim is to estimate the molecular properties, with an accuracy comparable with experiment^44^.

### Materials used

The investigation utilized molecular dynamics (MD) and Monte Carlo simulation techniques to create mixed matrix membranes (MMMs) using Polysulfone (PSF), Polydimethyl Siloxane (PDMS), and zinc oxide (ZnO) nanoparticles.

PDMS is a rubbery polymer with exceptional gas permeability, super hydrophobic properties, and excellent mechanical and chemical stability^57,58^. PSF is a glassy polymer that performs well in separating CO_2_^59^. ZnO is a common nanoparticle with attractive attributes such as low cost, good chemical, electrical, and mechanical properties, and a high surface-to-volume ratio compared to other nanoparticles^60^. ZnO nanoparticles are also a great option for CO_2_ adsorption due to their inherent affinity^61–63^.

### Theory and simulation procedure

Combination of significant properties of NPs with the natural features of polymers undoubtedly improves the physical and transport properties of novel MMMs^64^. In present article, the gas transport behavior of PSf polymer blended with PDMS, and loaded with ZnO NPs has been investigated It is worth pointing that, solution-diffusion is the dominant mechanism of dense membranes regarding the transport behavior and associated diffusion and solubility coefficients^56,65^. Different parameters such as the interactions of polymer-gas molecules, gas–gas has much of a role to play in altering diffusivity and solubility coefficients. The permeability and selectivity values can be calculated by Eqs. (1) and (2), respectively.

1 $${P}_{A}={D}_{A \times }{S}_{A},$$

2 $${\alpha }_{A/B}=\left(\frac{{D}_{A}}{{D}_{B}}\right)\times \left(\frac{{S}_{A}}{{S}_{B}}\right),$$

where ${D}$ is the diffusivity coefficient, $S$ is the solubility coefficient and ${\alpha }_{A/B}$ is the selectivity of gas A/B^64,66,67^. Generally, the selectivity can be defined as the permeability of one component over the other one which literally indicates the competence of each gas molecules.

Regarding the simulation process, Materials Studio software package from Accerlys Inc version 6.0 and COMPASS II force field was utilized to construct raw materials and conduct all the simulation steps. GCMC and MD are the two most oftenly utilized methods to determine the solubility and diffusivity coefficients, respectively. Various MMMs were simulated using different weight percent of PSf, PDMS and ZnO NPs. Some analysis like FFV, T_g_, and XRD have been applied to determine the structural features and properties of the constructed membranes. Adsorption isotherms and Mean Square Displacement (MSD) graphs were additionally utilized to estimate both solubility and diffusivity coefficients, respectively. To this end, the present molecular simulation study (at microscopic level) prognosticated the gas separation properties of all constructed MMMs.

### MMM construction

**Figure 1:**
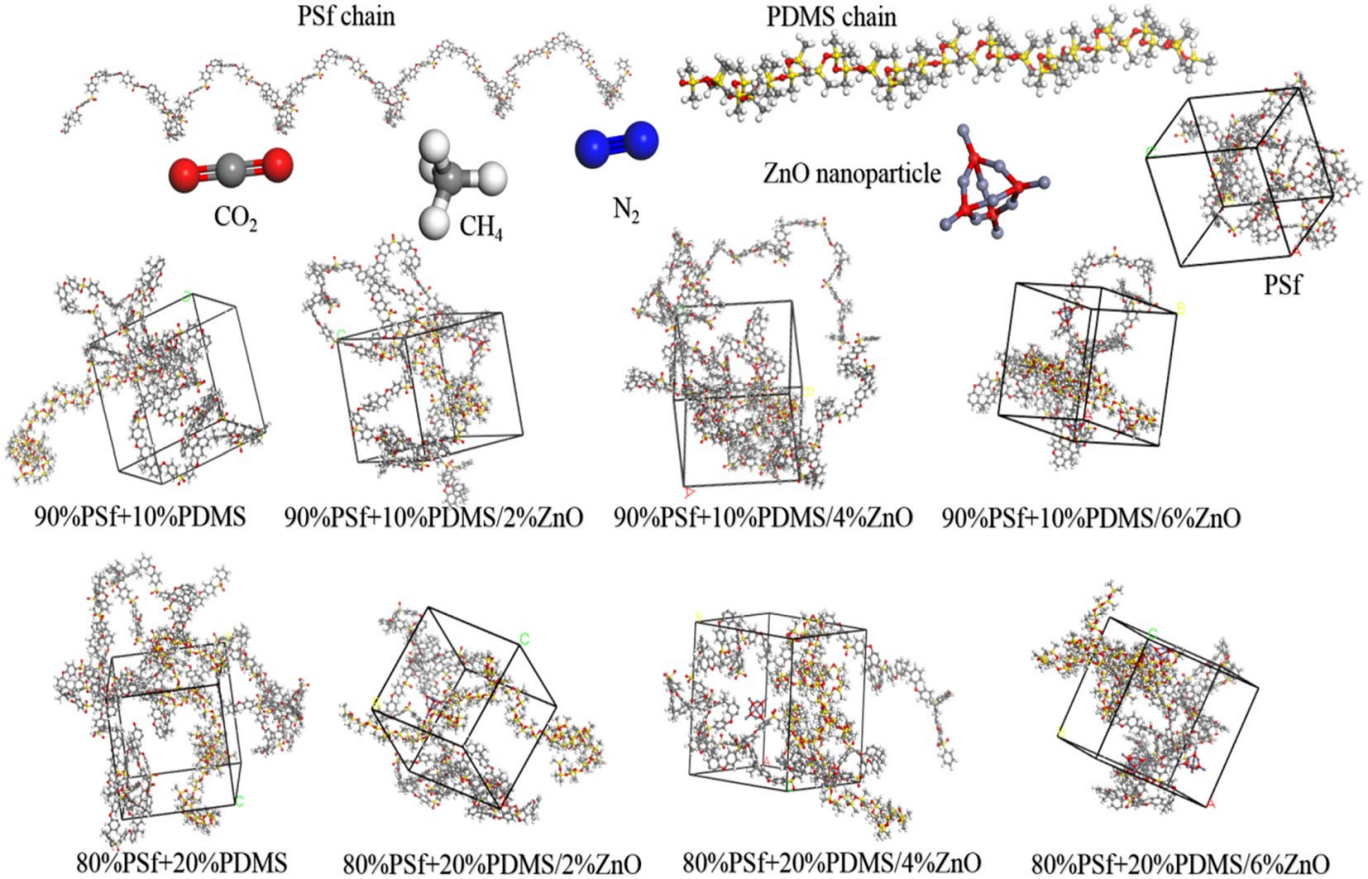
Molecular structures of simulated PSf, MMMS, ZnO nanoparticle, CH_4_, CO_2_, and N_2_ molecules at 298 K.

The periodic cells were simulated employing PSf and PDMS polymers chain with 10 chain length. Clearly, 10 and 20 wt% of PDMS was blended with PSf polymer to evaluate the effect of polymer blending. Additionally, the simulated cells were cubic in shape and sized between 30–40 Å, depending on the amount of materials loaded. The blended polymers were loaded by 2, 4 and 6 wt% of ZnO NPs. Hence, various MMMs were simulated. The ZnO NP was constructed in a 5 Å cubic form. Figure 1 indicates the periodic cells and raw materials^68^. Constructed materials, polymer chains and NP were also optimized from the energy and geometry perspective. It was chosen for the amorphous module to create 5 output frames; whereas 0.7 g cm^−3^ (at 298 K) was the selected value for the initial density. Finally, the obtained amorphous cells blended by 10 and 20 wt% of PDMS and dissimilar ZnO loading were acquired. Table 1 indicates the 9 different simulated membranes and their appointed names.

**Table 1:** The appointed names of simulated membranes.

No.	Membranes names
1	PSf
2	90%PSf + 10%PDMS
3	90%PSf + 10%PDMS/2%ZnO
4	90%PSf + 10%PDMS/4%ZnO
5	90%PSf + 10%PDMS/6%ZnO
6	80%PSf + 20%PDMS
7	80%PSf + 20%PDMS/2%ZnO
8	80%PSf + 20%PDMS/4%ZnO
9	80%PSf + 20%PDMS/6%ZnO

Forcite module was applied to optimize the simulated membranes. The non-equilibrium energy was eliminated by choosing smart method for better convergence. The obtained configurations were considered and the one showing the lowest level of energy was selected. The annealed procedure performing in the range of 298 and 500 k at a 5-cycle process was applied in an NPT run. Then, a 4000 ps-NPT run was implemented over the selected configuration to attain the final and experimental density. Additionally, in order to equilibrate the membrane structure with the experimental density, a 1000 ps-NVT run was conducted. Simultaneously, all gas molecules were simulated and then optimized by Forcite module. All the experiments were conducted at 298 K. Additionally, in order to control the temperature at the designated heating temperature and pressure of 1 atm, the simulation utilized the Nose thermostat with a Q ratio of 0.01 and the Berendsen barostat with a decay constant of 0.1 ps. Herein, the COMPASS II force field, along with atom-based electrostatic and van der Waals summation methods were selected with a fine cutoff distance of 12.5 Å. Figure 1 demonstrate the final configurations of optimized MMMs.

## Results and discussion

### Simulation methods

#### Fractional free volume (FFV)

The FFVs of simulated periodic cells as a membrane have been deliberated using spherical probe with Connolly radius of 0 nm. FFV values can be calculated by Eq. (3) as follows:^49,56,69,70^.

3 $$f=\frac{{V}_{s}-{1.3V}_{w}}{{V}_{s}},$$

where V_w_ and V_s_ are van de Waals and specific volumes, respectively. It is worth pointing that, the polymer chains’ occupied volume is usually 1.3 times greater than their van der Waals volume.

With reference to the results summarized in Table 2, incorporation of PDMS and ZnO NP into the membrane matrix resulted in higher FFV. Also, 80%PSf + 20%PDMS membrane loaded with 6 wt% ZnO indicated the highest FFV value. Moreover, FFV value increased from 17.1 to 20.8 because of more NP content introduced into the polymer matrix for PSf and 80%PSf + 20%PDMS, respectively. In general run of things, more nanomaterial loading more voids creation between polymer chains which takes place with greater d-spacing values. The special structure of ZnO undoubtedly enhanced the polymer chain distances and caused more fractional volume in polymer matrix. According to the FFV results, the effect of ZnO loading in PSf membrane matrix is obvious which follows an increasing trend starting from 17.01 to 20.1. The resulted FFV data are summarized in Table 2.

**Table 2:** The FFV values of all simulated membranes.

Samples	% of nanomaterial			
	0	2	4	6
PSf	17.1	18.3	19.6	20.1
90%PSf + 10%PDMS	17.3	18.7	19.7	20.3
80%PSf + 20%PDMS	17.6	19.2	20.1	20.8

#### Glass transition temperature (T_g_)

**Figure 2:**
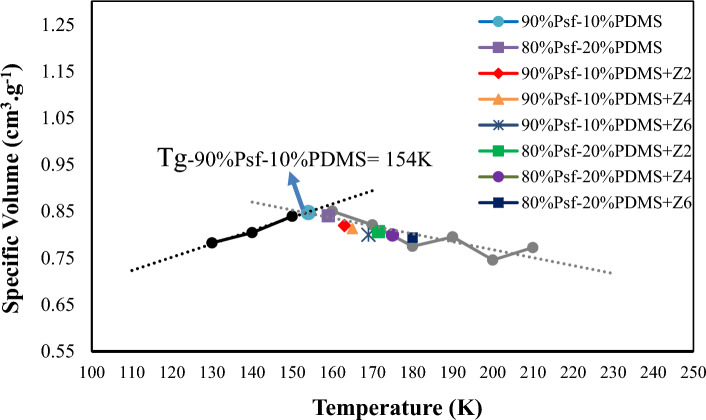
The calculated T_g_ of simulated samples.

T_g_ is a transition temperature that estimates the change in material state from a glassy state to a rubbery state that happens in amorphous polymers. As Fig. 2 indicates, the T_g_ of the constructed MMMs has displayed an increasing trend with ZnO loading. Also, the effect of loading 10 and 20 wt% of PDMS into the PSf polymer matrix is considerable. Which proves the resulted polymers stemming from combination of PSf and PDMS loaded ZnO tend to higher T_g_ values. The glassy temperature of polymer blends can be calculated from Fox equation as below^56,71–76^:

4 $$\frac{1}{{T}_{g-mix}}=\sum_{0}^{i}\frac{{\omega }_{i}}{{T}_{g-i}}.$$

Here ${T}_{g-mix}$ and ${T}_{g-i}$ are the T_g_ of the mixture/copolymer and of the components, respectively, and ${\omega }_{i}$ is the mass fraction of component i. It was observed that increase in ZnO content made slight changes in T_g_, which support the suggestion of no evident interaction between PDMS and ZnO NPs. In this subject, this happening can be attributed to the limited movement of the polymer backbone arising from PSF/PDMS–ZnO interactions. Figure 2 shows the calculated T_g_ for all simulated membranes.

#### X-ray diffraction (XRD)

**Figure 3:**
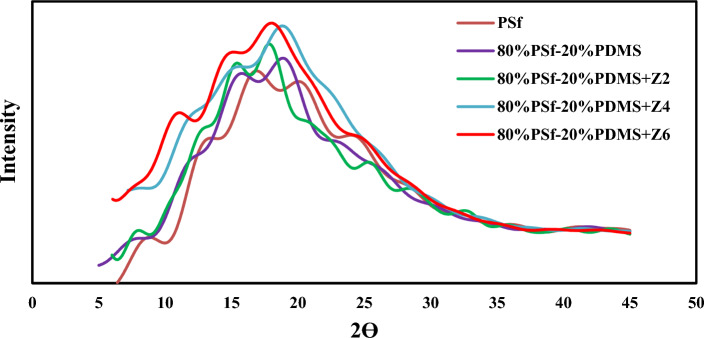
The scattering patterns of the PSF and 80% PSf + 20% PDMS, 80% PSf + 20% PDMS/2% ZnO, 80% PSf + 20% PDMS/4% ZnO and 80% PSf + 20% PDMS/6% ZnO.

As can be seen in Fig. 3 presenting the scattering diffraction patterns of the MMM, the maximum peaks are usually considered more significant than other patterns because of the possibility of calculating the d-spacing values based on Bragg’s equation $\left(d=\frac{\lambda }{2} {\text{sin}}\theta \right)$. This equation explains the intersegmental distances between polymer back bones^68,77^. By comparing the XRD patterns of simulated membranes, it can be concluded that the main peak of each sample is 2θ = 15°–20° and with increment of ZnO content, the main peak gets sharper and mainly locates in lower 2θ. For instance, the value of 2θ of pure PSf is around 17.00 ± 0.5%, while by adding 2 wt% of ZnO to the matrix, this value changes significantly and gets sharper. The presence of ZnO in PSf results in the expansion of the distance among PSf chains, and this fact has been shown by comparing d-spacing of each membrane. D-spacing of simple PSf is reported as 4.74 Å. Also, the d-spacing of 80%PSf + 20%PDMS, 80%PSf + 20%PDMS/2%ZnO, 80%PSf + 20%PDMS/4%ZnO and 80%PSf + 20%PDMS/6% ZnO are reported as 4.85 Å, 5.04 Å, 5.27 Å, and 5.46 Å, respectively. Wang et al.^78^ calculated d-spacing of pure PSf membrane about 5.2 Å. In another study, Golzar et al.^77^ indicated that its d-spacing was around 4.98 Å. Overall, this value is comparable to the simulated results of current study which both show consistency in approach.

#### Density

**Figure 4:**
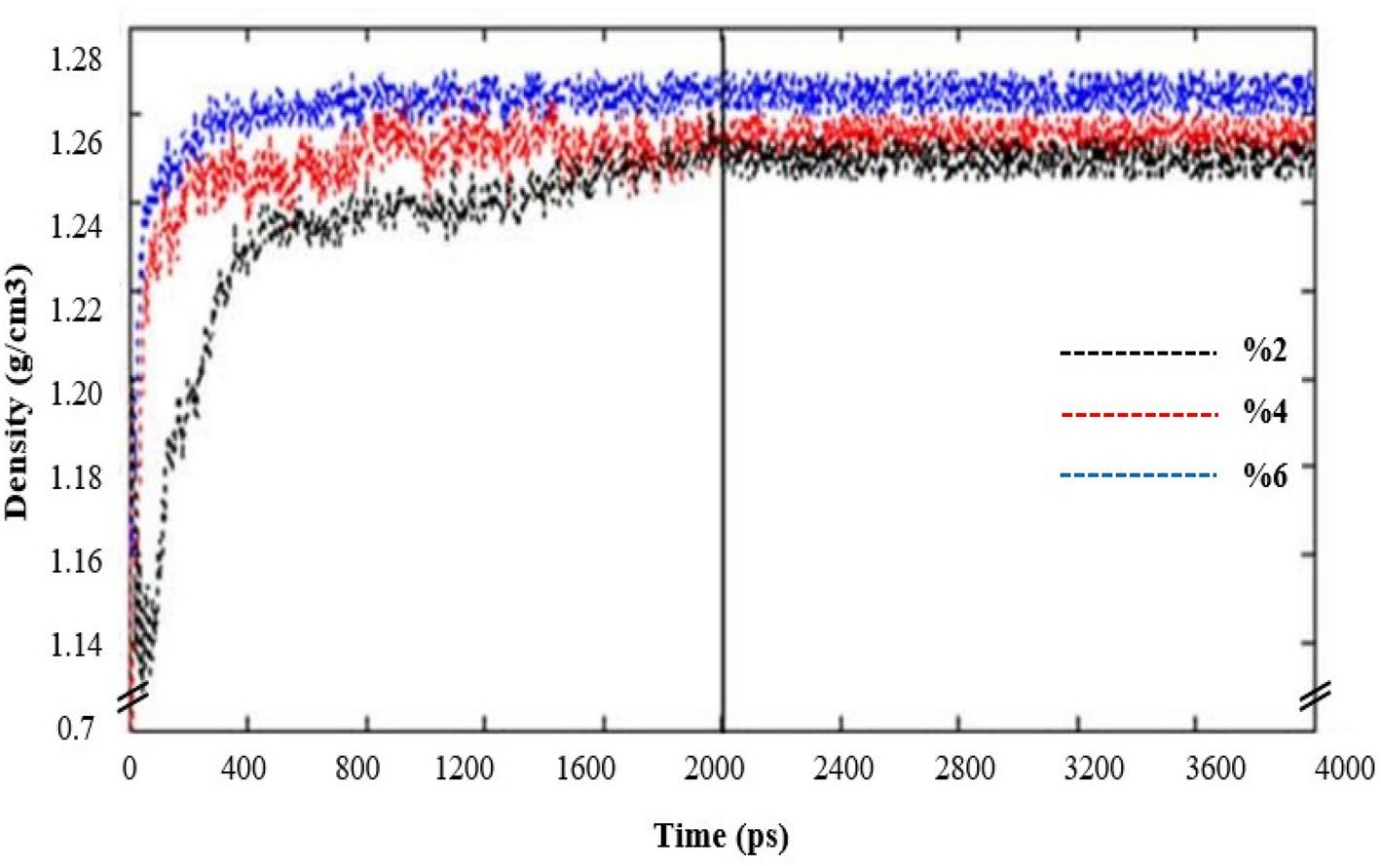
The acquired average densities of 80% PSf + 20% PDMS membranes loaded by 2, 4, and 6 wt% ZnO*.*

In current molecular simulation study, after selecting the density with initial value of 0.7 g cm^−3^, the process of constructing MMMs was applied. Also, Fig. 4 indicates the density graph of 80%PSf + 20%PDMS membranes loaded by 2, 4, and 6 wt% ZnO at 4, 8, 12 and 16 bar. It was observed that, the number of loaded PSf, PDMS chains and the amount of ZnO NPs in the polymer matrix directly effect of the density values of simulated membranes with various and different length as Table 3 summarized the acquired average densities of simulated membranes. It is noteworthy that, NPT runs have been applied to attain the actual density of each system which definitely modifies the density and dimensions of each cell. Consequently, the density of cells started to increase by a considerable reduction in cell length. Figure 4 indicates that the membrane density increased along with run time, whereas after 2000 ps a plateau was reached for the rest of the operating time. Therefore, the adequacy of the 4000 ps-NPT run was confirmed for reaching the equilibrium state.

**Table 3:** The acquired densities of all simulated simple and MMMs at 298 K and different pressure (4, 8, 12, and 16).

Pressure (bar)	Samples	% of nanomaterial			
		0	2	4	6
4	PSf	1.19	1.21	1.22	1.24
4	90%PSf + 10%PDMS	1.205	1.22	1.23	1.25
8	90%PSf + 10%PDMS	1.19	1.20	1.206	1.235
12	90%PSf + 10%PDMS	1.188	1.197	1.204	1.231
16	90%PSf + 10%PDMS	1.186	1.197	1.201	1.229
4	80%PSf + 20%PDMS	1.21	1.24	1.25	1.26
8	80%PSf + 20%PDMS	1.207	1.234	1.242	1.257
12	80%PSf + 20%PDMS	1.202	1.230	1.239	1.253
16	80%PSf + 20%PDMS	1.195	1.226	1.236	1.25

#### Mean square displacement (MSD)

Based on the Verlet algorithm, the motion equations with 1 fs time step was employed as a promising approach to ensure the energy conservation as well as predicting the self-diffusion coefficient $({D}_{i})$. Equation (5) under the name of the Einstein equation indicates the calculation of the self-diffusivity of component $(i)$:

5 $${D}_{i}=\frac{1}{6}\frac{d}{{d}_{t }}{lim}_{t\to \infty }MSD\left(t\right).$$

**Figure 5:**
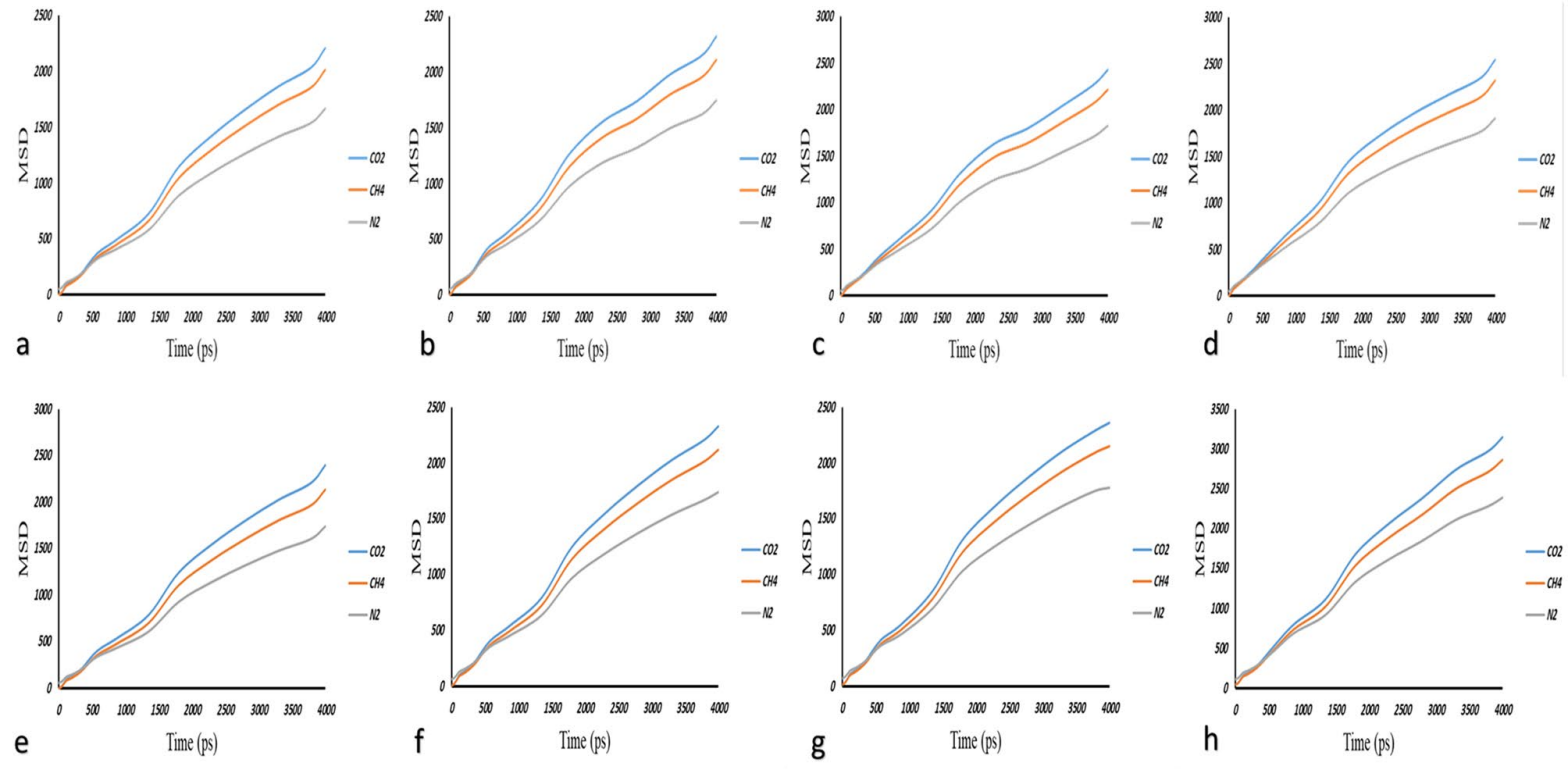
MSD of CO_2_, N_2_, CH_4_, gases at 298 K and 4 bar pressure within: (**a**) 90% PSf + 10% PDMS, (**b**) 90% PSf + 10% PDMS/2% ZnO, (**c**) 90% PSf + 10% PDMS/4% ZnO, (**d**) 90% PSf + 10% PDMS/6% ZnO, (**e**) 80% PSf + 20% PDMS, (**f**) 80% PSf + 20% PDMS/2% ZnO, (**g**) 80% PSf + 20% PDMS/4% ZnO, and (**h**) 80% PSf + 20% PDMS/6% ZnO.

Based on Eq. (5), CO_2_, N_2_ and CH_4_ gases start penetrating within the membrane relying on the diffusion mechanism happening in a pico-second. It is worth to note that, this motion relation is proportional to ${t}^{x}$ function when the initial condition (*x* < 1) applies^79^. To calculate ${D}_{i}$ of all gases in simple PSf and all MMMs, three different gas molecules (CO_2_, N_2_, CH_4_) with optimized geometries and minimized energies were inserted into the simulated membrane. The final configurations indicated the minimum energy. Then, the MSD results were evaluated and consequently the ${D}_{i}$ was calculated. These data were the results of three consecutive experiments as an average which were reported in Table 4. By taking a look at Table 4, it becomes obvious that the ${D}_{i}$ of gas molecules within the constructed membranes increases by higher loading of ZnO stemming from higher FFV and more free paths for gas diffusion. The same trend was observed for loading 10 and then 20 wt% of PDMS. To make sure that the MD simulation results are reliable, Fig. 5 revealed that the slope of Log (MSD) vs. Log (time) tend to reach unit^80^. As can be seen in this figure, the amount of MSD for CO_2_ gas is higher than N_2_ and CH_4_, respectively, because of the linear structure of this gas which accelerates and increases the transfer diffusion through the MMMs compare to methane, which has a Tetrahedron structure.

**Table 4:** The ${D}_{i}$ coefficients of CO_2_, CH_4_, and N_2_ through all simulated simple membrane and MMMs at 298 K and 4, 8, 12 and 16 bar pressure.

Pressure (bar)	Samples	% of nanomaterial											
		0			2			4			6		
		CO_2_	CH_4_	N_2_	CO_2_	CH_4_	N_2_	CO_2_	CH_4_	N_2_	CO_2_	CH_4_	N_2_
4	PSf	0.028	0.011	0.024	0.032	0.0135	0.0262	0.0396	0.0212	0.0336	0.0465	0.0253	0.0361
4	90%PSf + 10%PDMS	0.031	0.0143	0.0262	0.03683	0.017	0.028	0.039	0.0238	0.029	0.0417	0.028	0.0328
8	90%PSf + 10%PDMS	0.032	0.0151	0.027	0.039	0.018	0.029	0.041	0.0252	0.030	0.0442	0.029	0.034
12	90%PSf + 10%PDMS	0.033	0.015	0.028	0.040	0.018	0.0305	0.042	0.025	0.031	0.045	0.030	0.035
16	90%PSf + 10%PDMS	0.034	0.0160	0.029	0.041	0.019	0.031	0.043	0.026	0.032	0.046	0.031	0.036
4	80%PSf + 20%PDMS	0.031	0.014	0.026	0.037	0.017	0.028	0.039	0.024	0.029	0.042	0.028	0.033
8	80%PSf + 20%PDMS	0.033	0.015	0.028	0.039	0.0183	0.030	0.042	0.025	0.031	0.045	0.030	0.035
12	80%PSf + 20%PDMS	0.034	0.015	0.0291	0.040	0.018	0.031	0.043	0.026	0.032	0.046	0.031	0.036
16	80%PSf + 20%PDMS	0.035	0.016	0.029	0.042	0.019	0.032	0.044	0.027	0.033	0.047	0.032	0.037

#### Solubility coefficients

To calculate the solubility coefficient of each gas molecules within the simulated simple PSf and MMMs, GCMC was hired including the Metropolis method as a reliable task in Sorption module. Besides, adsorption isotherms is another task that can evaluate the effect of some experimental condition such as pressure and temperature on the solubility coefficient. Additionally, one of the other advantages of GCMC method is reaching to a better understanding of the sorption mechanism at the atomistic level. This sorption mechanism can be included some values such as regrowth, conformer rotation, translation and exchange. Notwithstanding, the Metropolis task involves a number of moves just like translation, rotation and exchange. Equation (6) indicates the acceptance probability as follows:

6 $${P}_{acc} \left(o\to n\right)=\mathrm{min}(1;\mathrm{exp}\left(-\left[ {U}_{\left(n\right)}+\frac{{U}_{\left(o\right)}}{{k}_{B}T}\right]\right),$$

where_,_ ${U}_{(n)}$, ${U}_{(o)}$ and ${k}_{B}$ are the potential energy of old state, the potential energy of new state, and Boltzmann's constant, respectively^81,82^.

**Figure 6:**
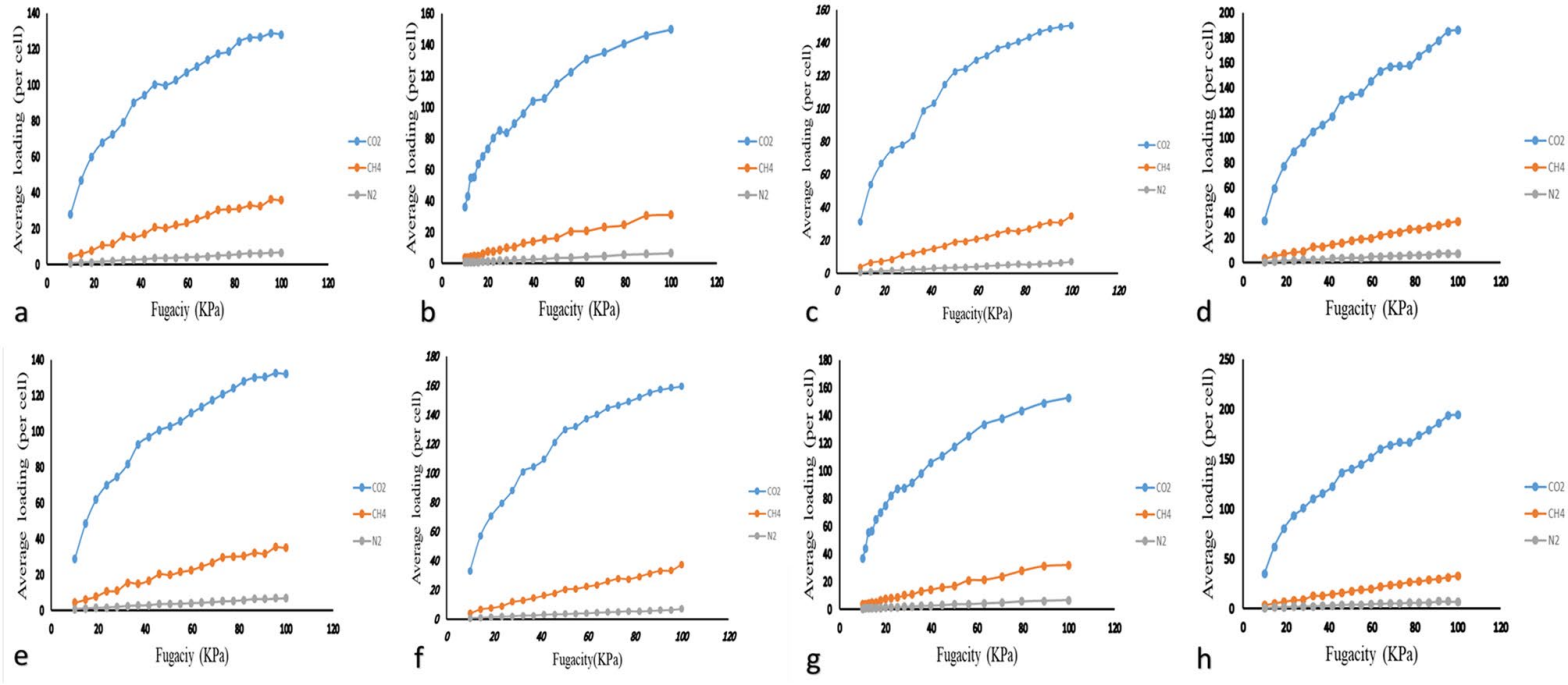
Adsorption isotherms of CO_2_, CH_4_, and N_2_ gas molecules for (**a**) 90% PSf + 10% PDMS, (**b**) 90% PSf + 10% PDMS/2% ZnO, (**c**) 90% PSf + 10% PDMS/4% ZnO, (**d**) 90% PSf + 10% PDMS/6% ZnO, (**e**) 80% PSf + 20% PDMS, (**f**) 80% PSf + 20% PDMS/2% ZnO, (**g**) 80% PSf + 20% PDMS/4% ZnO, and (**h**) 80% PSf + 20% PDMS/6% ZnO.

Generally, GCMC method works based on the trial insertion and deletion of molecules, in which, 10^5^ equilibrium steps and 10^6^ production steps^44^ were set to conduct the adsorption isotherm calculations. Equation (7) shows the probability of rejecting or accepting a new location for any gas as follows:

7 $$Probe=\mathrm{min}\left(1;\mathrm{exp}\left(\frac{-\Delta E}{KT}\pm ln\frac{{N}_{i } kT}{{f}_{i } V}\right)\right),$$

where $\Delta E$, ${f}_{i}$,${N}_{i}$, and $V$ can be defined as the difference of Van der Waals interaction and columbic interaction for two configurations, the fugacity, the number of molecules for component i, and the volume of amorphous cell, respectively^68,83^. To measure the solubility coefficient, the slope of adsorption isotherms represents was measured as Eq. (8) indicates^65,84^:

8 $$S={lim}_{p\to 0}\left(\frac{C}{P}\right),$$

where $P$ is the fugacity and $C$ represents the gas concentration. Figure 6 indicates the adsorption isotherm diagrams for all diffusing molecules across the simulated membranes.

Generally, various factors can directly affect on gas permeation such as FFV, crystallinity, pressure, temperature, and so on. In current simulation study, the structural features and separation properties of constructed membranes have been affected by different factors from different aspects. Inevitably, some of them had negative impact on gas separation performance while some others positively enhanced its performance. Therefore, the summation of all these positive and negative effects lead to a certain value for gas permeability. So, evaluating all these factors can be of great help to reach a better understand of the system.

As it is obvious in Table 5, CO_2_ solubility within the membranes is much greater than CH_4_ and N_2_. The main reason may be attributed to the fact that CO_2_ is an acid gas and PSf shows better affiliation with CO_2_. Also, Table 5 shows that, the solubility coefficients of pure gases through the membranes generally enhance with more ZnO content, which indicates the effect of presence of ZnO NPs in polymer matrix. Also, these figures experience an upward trend when the PDMS content increases. Although, the T_g_ experiences a slight increase, higher solubility coefficients convey this meaning that the membranes confronted with expanded amorphous region which provided the polymer matrix with a higher chance of adsorbing more gas molecules. To clarify, the increasing trend of measured slopes regarding the adsorption isotherms proves the gradual increase of the solubility coefficients of utilized gas molecules. On the other side, the acquired results of MSD analysis revealed that the diffusivity coefficients of each gas molecules experienced a gradual increase due to the presence of PDMS polymer chains and more pore and channels of created by ZnO NPs. According to the conducted experiments, the results of gas permeability have been evaluated exhaustively in the next section.

**Table 5:** The solubility coefficients of CO_2_, CH_4_, and N_2_ within the simulated membranes.

Pressure (bar)	Samples	% of nanomaterial											
		0			2			4			6		
		CO_2_	CH_4_	N_2_	CO_2_	CH_4_	N_2_	CO_2_	CH_4_	N_2_	CO_2_	CH_4_	N_2_
4	PSf	1.29	0.065	0.021	1.34	0.071	0.023	1.335	0.074	0.026	1.25	0.072	0.026
4	90%PSf + 10%PDMS	1.35	0.071	0.025	1.404	0.074	0.029	1.397	0.077	0.0325	1.29	0.076	0.0323
8	90%PSf + 10%PDMS	1.431	0.075	0.026	1.488	0.0784	0.030	1.480	0.081	0.034	1.36	0.0805	0.0342
12	90%PSf + 10%PDMS	1.471	0.077	0.027	1.530	0.080	0.031	1.522	0.083	0.0354	1.406	0.082	0.035
16	90%PSf + 10%PDMS	1.512	0.079	0.028	1.572	0.082	0.032	1.5646	0.086	0.0364	1.444	0.08512	0.036
4	80%PSf + 20%PDMS	1.378	0.072	0.025	1.433	0.075	0.029	1.426	0.078	0.033	1.317	0.077	0.032
8	80%PSf + 20%PDMS	1.461	0.076	0.027	1.519	0.080	0.031	1.511	0.083	0.035	1.396	0.0822	0.034
12	80%PSf + 20%PDMS	1.502	0.079	0.027	1.562	0.082	0.032	1.554	0.085	0.036	1.435	0.084	0.035
16	80%PSf + 20%PDMS	1.543	0.081	0.028	1.605	0.084	0.033	1.597	0.088	0.0371	1.4751	0.086	0.036

## Gas permeability and perm-selectivity

In general, the permeability can be explained as the multiplication of solubility and diffusivity coefficients. In this section, the permeability of three pure gas molecules were calculated to thoroughly investigate the performance of simulated membranes. In this regard, two loadings of PDMS and 4 different loading of ZnO NPs have been incorporated into the PSf matrix. So, it can be perceived that NPs loading is another influential factor affecting the gas permeability.

Notably, as mentioned before all raw materials have been optimized geometrically and minimized in aspect of energy level. Also, all simulation practices were performed at thermodynamic equilibrium state. A 1000-ps NVT and 4000-ps NPT MD runs were conducted to eliminate the non-equilibrium states and reach the final density. It should be noted that, Nose–Hoover thermostat was chosen as the temperature controller these MD runs.

**Figure 7:**
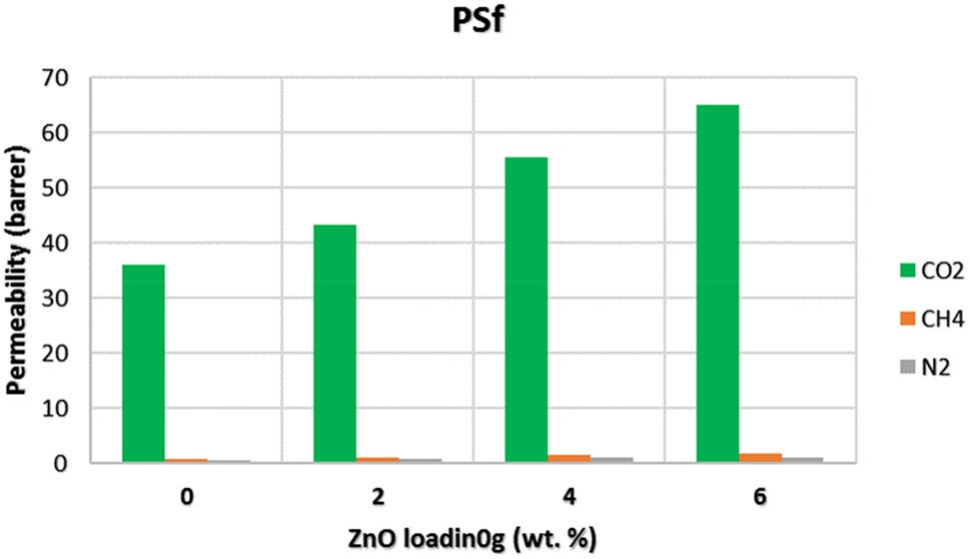
The effect of ZnO loading on simple PSf membrane.

All three gas molecules were inserted into the simulated MMM to measure the ${D}_{i}$. This coefficient can be calculated as the slope of MSD graph. The reason that validates the obtained results is that the slope of Log (MSD) vs. Log (time) diagram for all gases unify^85^. On the other side, having used the GCMC method, the solubility coefficients of all gas molecules were computed. Figure 7 illustrates the effect of ZnO loading on gas permeability through simple PSf membrane.

**Figure 8:**
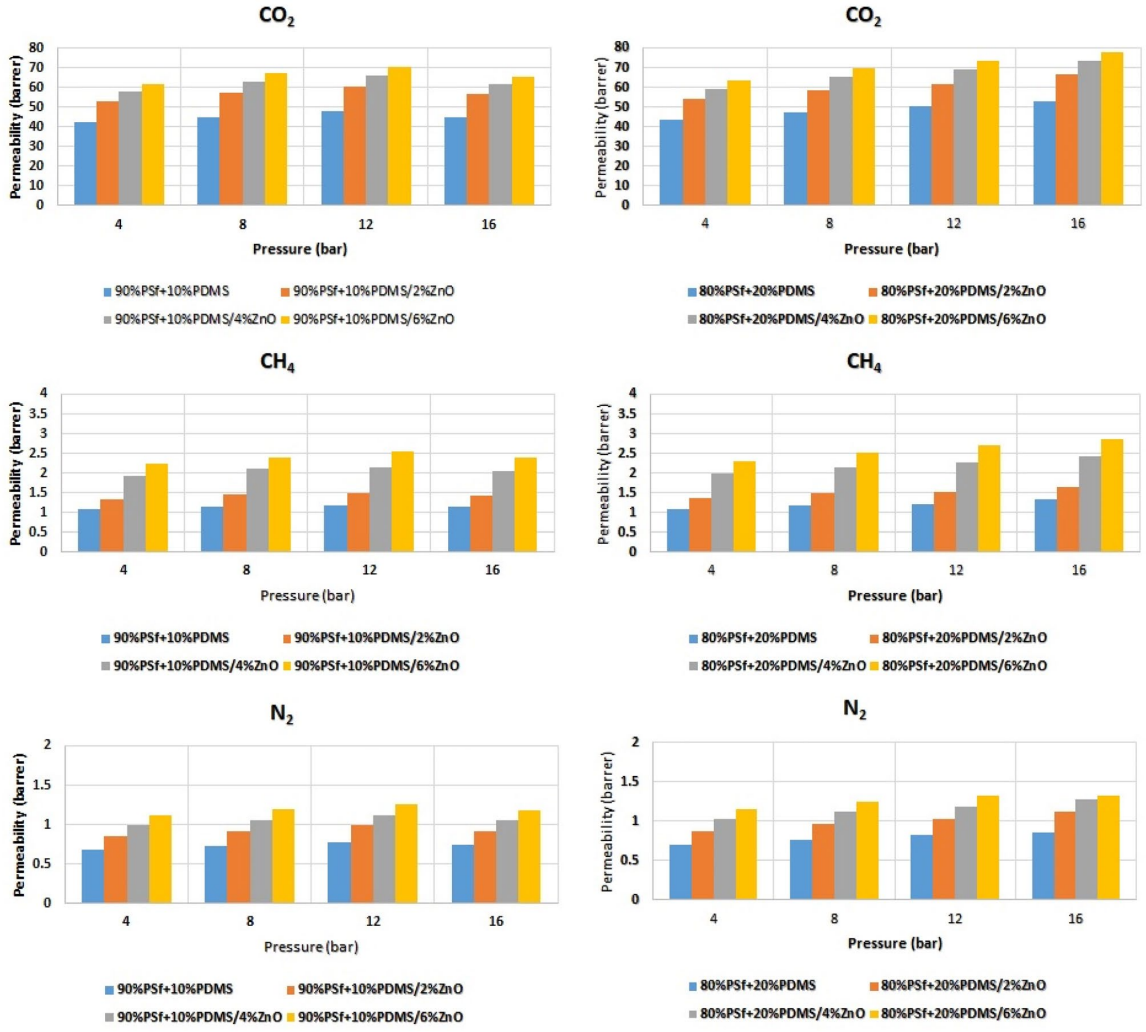
The effect of ZnO loading and PDMS blending on gas permeabilities.

As can be seen from Fig. 7, CO_2_ showed the highest permeability over two other gases. CH_4_ permeability is considerably greater than N_2_ permeability. Besides, it is obvious that, loading more ZnO content led to greater permeabilities of all gasses. The other simulated MMMs were tested by gas permeability and the effect of ZnO loading and PDMS blending were considered. Figure 8 indicates clearly the effect of these parameters. A brief look at Fig. 8 indicates that adding more ZnO content due to providing higher d-spacing and expanded distances between polymer chains generally leads to higher permeability values. Although, the effect of NPs is considerable, in some cases loading 6 wt% of ZnO resulted in lower permeability compare to 4 wt% which may stem from agglomeration of NPs playing a negative role against permeability. Additionally, by applying more operational pressure (4, 8, 12, and 16 bar), permeability of all three gases increases^71^. On the other side, the perm-selectivity of membranes are listed in Table 6. It is clear that ZnO loading negatively effects on CO_2_/CH_4_ selectivity, while PDMS blending increases its selectivity. Besides, CO_2/_N_2_ selectivity followed the same trend in both ZnO loading and PDMS blending.

**Table 6:** The effect of feed pressure, ZnO loading and PDMS blending on gas perm-selectivity.

Pressure (bar)	*S*ample	% of nanomaterial							
		0		2		4		6	
		CO_2_/N_2_	CO_2_/CH_4_	CO_2_/N_2_	CO_2_/CH_4_	CO_2_/N_2_	CO_2_/CH_4_	CO_2_/N_2_	CO_2_/CH_4_
4	PSf	60.2	46.24	56.85	43.24	50.91	34.05	55.71	33.78
4	90%PSf + 10%PDMS	61.05	39.38	61.23	39.52	58.09	29.87	54.98	27.37
8	90%PSf + 10%PDMS	61.15	38.50	63.05	39.52	59.04	29.66	56.22	28.01
12	90%PSf + 10%PDMS	60.81	39.97	61.05	40.54	58.52	30.63	55.60	27.57
16	90%PSf + 10%PDMS	60.49	38.60	61.56	39.36	58.05	30.15	55.26	27.27
4	80%PSf + 20%PDMS	61.52	40.23 (38.22)*	61.51	39.70	58.09	29.62	55.04	27.57
8	80%PSf + 20%PDMS	60.81	39.97 (45.57)*	60.51	38.87	58.52	30.63	55.60	27.57
12	80%PSf + 20%PDMS	60.28	41.18	60.06	40.54	58.05	30.15	55.26	27.27
16	80%PSf + 20%PDMS	61.77	39.79	59.62	40.86	57.09	30.23	58.57	27.30

*The experimental value reported in the literature^86^.

The before mentioned trends can be attributed to the ZnO nature and structure which tends to let all the three gases pass freely within the membrane. Also, the PDMS blending enhanced the MMMs performance resulted in better gas separation. The increasing feed pressure was considered as a positive effect on perm-selectivity which moderately changed their performance. The results obtained from simulation study were compared with the experiments for the perm-selectivity within PSF/PDMS composite membrane, without ZnO NPs^86^. It was concluded that both simulation and experimental results are in good agreement.

## Comparison with the literature

The effectiveness of MMMs for technical uses is determined by two primary factors: selectivity and permeability. In this study, the selectivity of gas pairs was compared to outcomes from earlier research and the compiled information is listed in Table 7 with corresponding references. The results indicate that the MMMs developed in this investigation have superior selectivity values for CO_2_/CH_4_ and CO_2_/N_2_ separation compared to previously studied membranes. The PSf/PDMS–Nano ZnO MMM exhibited significant potential for industrial applications such as natural gas sweetening or biogas purification and warrants further exploration.

**Table 7:** Literature data for MMMs for comparison of CO_2_/N_2_ and CO_2_/CH_4_ selectivities.

Membrane type	Condition	P_CO2_	${\text{CO}}_{2}/{\text{CH}}_{4}$	${\text{CO}}_{2}/{\text{N}}_{2}$	References
Pebax-1657/PVC	10 bar	3 GPU	25.8	55.5	^87^
PSf/4-PVP/SR	14 bar/25 °C	92 GPU	29	–	^88^
Matrimid^®^/ZIF-8	10 bar/35 °C	23 GPU	23	27	^89^
Ultem^®^/ZIF-8	6.89 bar/25 °C	18 GPU	–	44	^90^
PI/Cu_3_(BTC)_2_	10 bar/25 °C	32 GPU	9	6	^91^
Ultem^®^/zeolites	1.6 bar/35 °C	6.2 GPU	44	30	^92^
Pristine Pebaxs-1657	3.75 bar/25 °C	287 GPU	14	34	^93^
PEBA-PEG-b-PPFPA/PDMS/PAN	3.5 bar/35 °C	1864 Barrer	–	22	^94^
This work	4–16 bar/25 °C	36.12–78.02 Barrer	27.27–46.24	50.91–63.05	

*ZIF-8* zeolitic imidazole framework 8, *Ultem*^*®*^ polyetherimide, *PI* Polyimid, *PEG-b-PPFPA* poly(ethylene glycol)-block-poly(pentafluoropropyl acrylate), *PVC* polyvinyl chloride, *4-PVP* poly (4-vinylpyridine), *SR* silicon rubber.

GPU (1 GPU = 1 × 10^−6^ cm^3^ (STP)/(cm^2^ s cmHg)).

## Robeson’s upper bound

**Figure 9:**
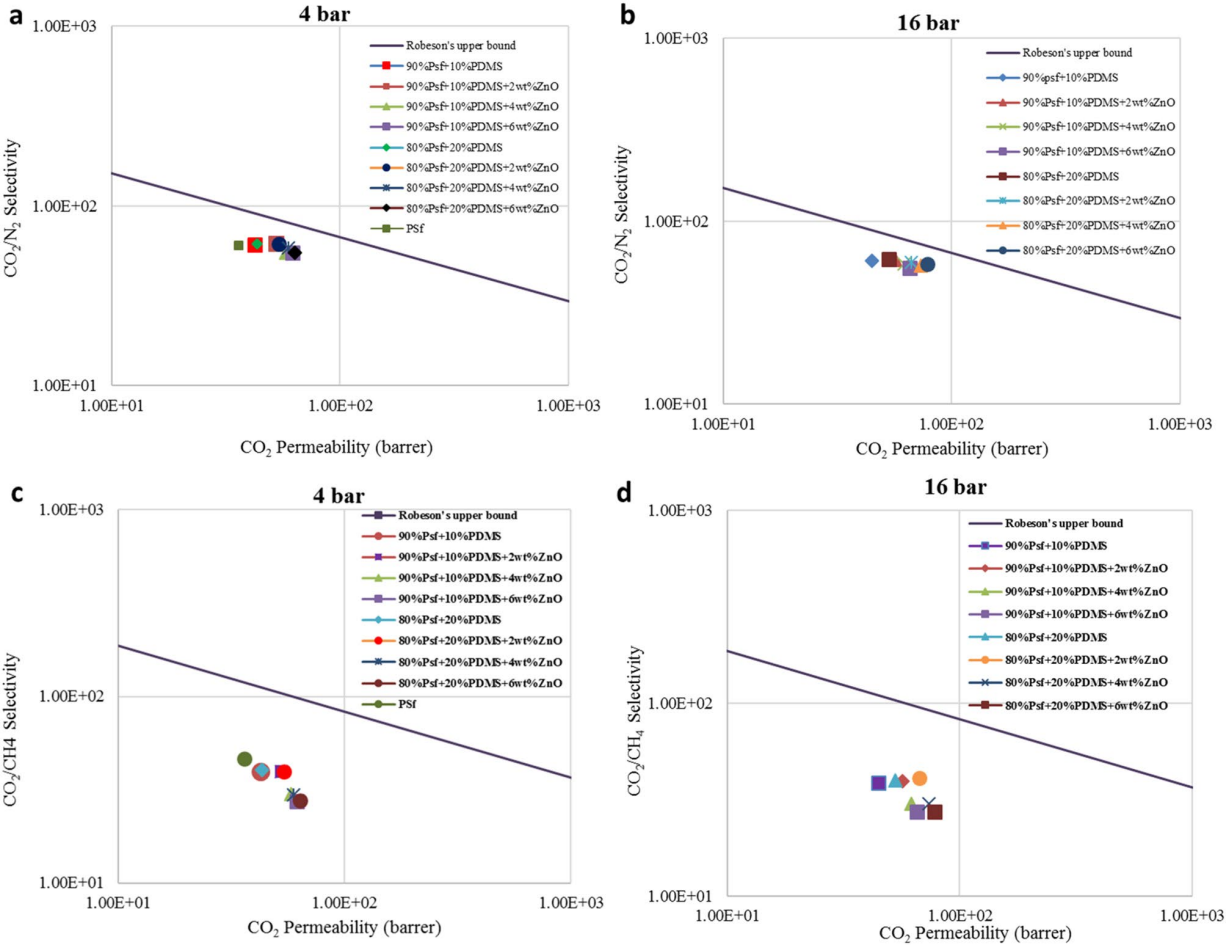
Robeson’s upper bound for CO_2_/N_2_ at (**a**) 4 bar and (**b**) 16 bar, and CO_2_/CH_4_ at (**c**) 4 bar and d) 16 bar (T = 298 K).

The gas separation performance of simulated periodic cells were examined by Robeson’s upper bound^14^ which is plotted for the selectivity vs. permeability of gas pairs of N_2_, CH_4_, and CO_2_. This plot indicates the acquired data based on the selectivity vs. permeability of the simulated membranes. What perceived from this plot is that which membranes demonstrated more appropriate separation performance compare to the industry standards^68,56^. In other words, closer points to the Robeson’s upper bound proclaim that those points associated with membrane have better separation performance. Figure 9 indicates the Robeson’s Upper Bound for CO_2_/CH_4_ and CO_2_/N_2_ at 4 and 16 bar. A brief look at the Fig. 9 shows that the simulated membranes had better CO_2_/N_2_ separation performance than CO_2_/CH_4_. This result is attributed to the fact that, CH_4_ showed double as the N_2_ permeability. Moreover, the membranes loaded by 4% wt% of ZnO had better performance than others in which 20 wt% PDMS blended membrane were moderately better than 10 wt. % blended ones.

## Conclusion

The permeability of some pure gas molecules (e.g., CO_2_, N_2_ and CH_4_) within sveral suggested MMMs were predicted using molecular simulation methods. In this regard, Psf polymer matrix was loaded by various content of ZnO NPs and blended 10 and 20 wt% of PDMS. Also, their performance was evaluated using MS by some structural and transport analysis. T_g_ and FFV results illustrated that the loading ZnO NPs directly influenced the membrane matrix, which led to different gas permeability. Blending 10 and then 20 wt% PDMS clearly improved the membrane performance. In other words, higher perm-selectivity was achieved by more blended polymer. ZnO loading resulted in higher T_g_ and more rigid sections. However, it improved the FFV values. It was shown that the more pressure applied, the more permeability and selectivity values resulted. The T_g_ of the simulated MMMs experienced an increasing trend with increasing ZnO content, indicating that MMMs had more extended rigid region than unfilled membranes. The fact that the slopes of adsorption isotherms experienced an increasing trend proved that that the solubility coefficients of employed gas molecules soared gradually. With reference to the MSD analysis, it was obvious that ${D}_{i}$ of gas molecules changed gently because of higher pore and channels, higher pressure, the ZnO content. In conclusion, this simulation study reveals that, the PSf/PDMS polymer matrix membrane incorporated with ZnO NPs might be a fascinating MMM for separation of CO_2_/CH_4_/N_2_ mixtures in gas refineries plants.

The transferability of a simulation method used in a study to other mixed matrix membranes for gas separation depends on several factors, such as the molecular interactions between the gas molecules and the membrane material, as well as the structural properties of the membrane. In general, the transferability of a simulation method can be improved if the method has been validated using experimental data and if it accounts for the specific properties of the membrane material, such as pore size, surface area, and surface chemistry. Additionally, the simulation method should be able to capture the dynamic behavior of the gas molecules within the membrane, including adsorption and desorption processes, as well as diffusion and transport. However, it is important to note that even with a well-validated simulation method, the transferability of the results to other mixed matrix membranes may be limited due to the differences in the composition and structure of the membranes. Therefore, it is recommended to validate the simulation method for each specific membrane material and to account for any differences in the properties of the membrane when interpreting the results. Therefore, the results obtained in current article have been compared with experimental results as discussed in previous sections.

Finally, MS can be considered as a prospective and profitable tool to not only estimate the structural features and separation properties of polymeric structures but also to optimize the operating factors which undoubtedly enhance the membrane separation performance relying on their promising features.

## Data Availability

All data generated or analysed during this study are included in this published article.
